# Epidemiological investigation of fetal laterality disorders in multiple hospitals in coastal Southeastern China before and after the COVID-19 pandemic (2017–2024)

**DOI:** 10.3389/fmed.2026.1786245

**Published:** 2026-04-23

**Authors:** Biying Huang, Zongjie Weng, Qiuxia Jiang, Fa Chen, Guorong Lyu, Zhilan Wang, Jiansu Hu, Xiuqin Wu, Xianlian Su, Wen Ling, Qiumei Wu

**Affiliations:** 1 Department of Medical Ultrasonics, Fujian Maternity and Child Health Hospital, College of Clinical Medicine for Obstetrics & Gynecology and Pediatrics, Fujian Medical University, Fuzhou, China; 2 Department of Medical Ultrasonics, Quanzhou Maternal and Child Health Hospital (Quanzhou Children's Hospital), Quanzhou, China; 3 Department of Epidemiology and Health Statistics, School of Public Health, Fujian Medical University, Fuzhou, China; 4 Department of Medical Ultrasonics, The Second Affiliated Hospital of Fujian Medical University, Quanzhou, China; 5 Department of Medical Ultrasonics, Nanping First Hospital Affiliated to Fujian Medical University, Nanping, China; 6 Department of Medical Ultrasonics, Fuzhou First General Hospital Affiliated with Fujian Medical University, Fuzhou, China; 7 Department of Medical Ultrasonics, Mindong Hospital Affiliated to Fujian Medical University, Ningde, China; 8 Department of Medical Ultrasonics, Putian University Affiliated Hospital, Putian, China

**Keywords:** COVID-19 pandemic, epidemiological investigation, fetal laterality disorders, Fujian, SARS-CoV-2

## Abstract

**Background:**

Fetal laterality disorders are rare congenital anomalies including situs inversus, partial heterotaxy, and heterotaxy syndrome. They are associated with increased risks of conditions such as congenital heart defects and primary ciliary dyskinesia. Recent studies have suggested a rise in the incidence of situs inversus following the COVID-19 pandemic. The purpose of this study is to investigate trends in the incidence of fetal laterality disorders before and after the COVID-19 pandemic.

**Materials and methods:**

This study included all pregnant women who underwent fetal ultrasound examinations between January 1, 2017, and December 31, 2024, at 15 hospitals in various regions of Fujian Province, a coastal southeastern city in China. Regions included Eastern Fujian, Southern Fujian, Western Fujian, Northern Fujian, and Central Fujian. The annual incidence of fetal laterality disorders was observed, and the association between the COVID-19 pandemic and the incidence of these disorders was assessed.

**Results:**

The total number of deliveries across the 15 hospitals was 721,562. Between 2017 and 2024, 566 cases of fetal visceral laterality disorders were diagnosed by prenatal ultrasound, including 178 cases of complete situs inversus, 139 cases of partial situs inversus, and 249 cases of heterotaxy syndrome (HS). During the COVID-19 infection period, the incidence of fetal laterality disorders showed an increasing trend from 2020 to 2024 (*P* < 0.05). Further analysis by time period revealed that the incidence during the Dynamic Zero-COVID Period (2020–2022) was 6.10 per 10,000 live births (95% CI: 5.18–7.02), which was lower than that during the pre-pandemic period (7.83 per 10,000 live births, 95% CI: 6.89–8.77). In contrast, during the post-policy adjustment period (2023–2024), the incidence increased to 10.61 per 10,000 live births (95% CI: 9.07–12.15), which was 1.4 times that of the pre-pandemic period, with the most noticeable increase observed in situs inversus. Geographically, the number of cases of fetal laterality disorders was higher in Southern Fujian and Eastern Fujian than in other regions.

**Conclusion:**

This study suggests an increased incidence of fetal visceral laterality disorders during the COVID-19 pandemic. It provides insights for further related research.

## Introduction

Coronavirus disease 2019 (COVID-19), caused by infection with severe acute respiratory syndrome coronavirus 2 (SARS-CoV-2), has had a profound impact on global public health (1). The effects of COVID-19 on pregnancy are not yet fully understood. Studies conducted at the beginning of the COVID-19 pandemic provided growing evidence supporting placental SARS-CoV-2 transmission (2, 3) and its association with various adverse fetal outcomes, including preeclampsia, premature rupture of membranes, preterm birth, low birth weight, and stillbirth (4–6). However, there are few studies on fetal developmental anomalies potentially related to SARS-CoV-2 infection, and the specific consequences of infection at different gestational stages, particularly the potential impact on fetal development, have not been thoroughly examined.

Human laterality disorders are a heterogeneous group of conditions primarily caused by alterations in the position or orientation of thoracic and abdominal organs and great vessels along the left-right axis, including situs inversus and heterotaxy syndrome (7). Situs inversus includes situs inversus totalis (SIT; dextrocardia) and partial situs inversus (SIP; isolated levocardia), with an incidence of 1/6,000–8,000 (abnormal development of human laterality). Situs inversus is characterized by a mirror-image reversal of the visceral organs; when the entire anatomical left-right axis is neither normal nor mirror-reversed, the resulting phenotype is often referred to as ambiguous situs or heterotaxy (8). Heterotaxy refers to abnormal organ positioning on the left or right side, with normally paired asymmetric organs losing their asymmetry. Its incidence is approximately 1/10,000 (7). Although over 100 genes have been identified to be involved in the visceral lateralization process in animal models, only a few genes are considered potential candidates for causing left-right asymmetry defects in humans, including NODAL, PITX2, and genes associated with primary ciliary dyskinesia (PCD) (9, 10). Additionally, maternal environmental risk factors are also implicated, although evidence remains limited (9). In particular, whether prenatal exposure to viral infections can lead to laterality disorders remains unclear. Current reports are limited to exploring the relationship between the prevalence of fetal situs inversus and infection with the novel coronavirus, with inconsistent conclusions regarding the exact relationship with SARS-CoV-2 infection, and a lack of research on the association between heterotaxy syndrome, partial situs inversus, and SARS-CoV-2 infection (11).

Therefore, given the global prevalence of COVID-19, it is crucial to evaluate the relationship between SARS-CoV-2 infection and fetal laterality disorders. This study aims to compare the incidence of fetal laterality disorders, including situs inversus, partial situs inversus, and heterotaxy syndrome, in 15 hospitals in Fujian Province, a coastal southeastern city in China, between 2017 and 2024.

## Materials and methods

### Study design and data sourses

We retrospectively analyzed data from pregnant women with fetal laterality disorders during pregnancy and either received routine prenatal care between January 1, 2017, and December 31, 2024, in six regions of Fujian Province, a coastal southeastern city in China. The regions included Western Fujian, Central Fujian, Eastern Fujian, Southern Fujian, and Northern Fujian. A total of 15 hospitals were included: in Western Fujian, Sanming First Hospital, Longyan People's Hospital, and Longyan First Hospital; in Central Fujian, Affiliated Hospital of Putian University; in Eastern Fujian, Fujian Maternity and Child Health Hospital, Fuqing Maternity and Child Health Hospital, Fuzhou First Hospital, Fuding Hospital, Ningde Hospital, and Ningde Mindong Hospital; in Southern Fujian, Quanzhou Maternity and Child Health Hospital, Quanzhou First Hospital, and Zhangzhou Hospital; in Northern Fujian, Nanping Maternity and Child Health Hospital and Nanping First Hospital. The total number of deliveries across the 15 hospitals was 721,562. Cases diagnosed by ultrasound with fetal situs inversus, partial situs inversus, and heterotaxy syndrome were included in the study (*n* = 566). The annual number of live births at each hospital was also collected. The incidence was standardized per 10,000 live births by year of delivery. Based on the government's response strategy and epidemiological characteristics, the epidemic in China can be divided into multiple stages. Following the outbreak in early 2020, China implemented a series of containment measures, transitioning from the initial emergency response to normalized prevention and control by April 2020, and subsequently to the dynamic COVID-zero strategy from August 2021 onward. A major policy shift occurred in December 2022 with the announcement of “10 New Measures” to optimize COVID-19 response, followed by the downgrading of COVID-19 management from Class A to Class B on December 26, 2022, marking the beginning of the post-pandemic relaxation period (12). Therefore, the period from 2017 to 2024 was divided into three phases: 2017–2019 (pre-pandemic period), 2020–2022 (strict pandemic control and Dynamic Zero-COVID Period), during which the Dynamic Zero-COVID policy was maintained, and 2023–2024 (post-pandemic period), during which policies were adjusted and herd immunity was established.

**Figure 1:**
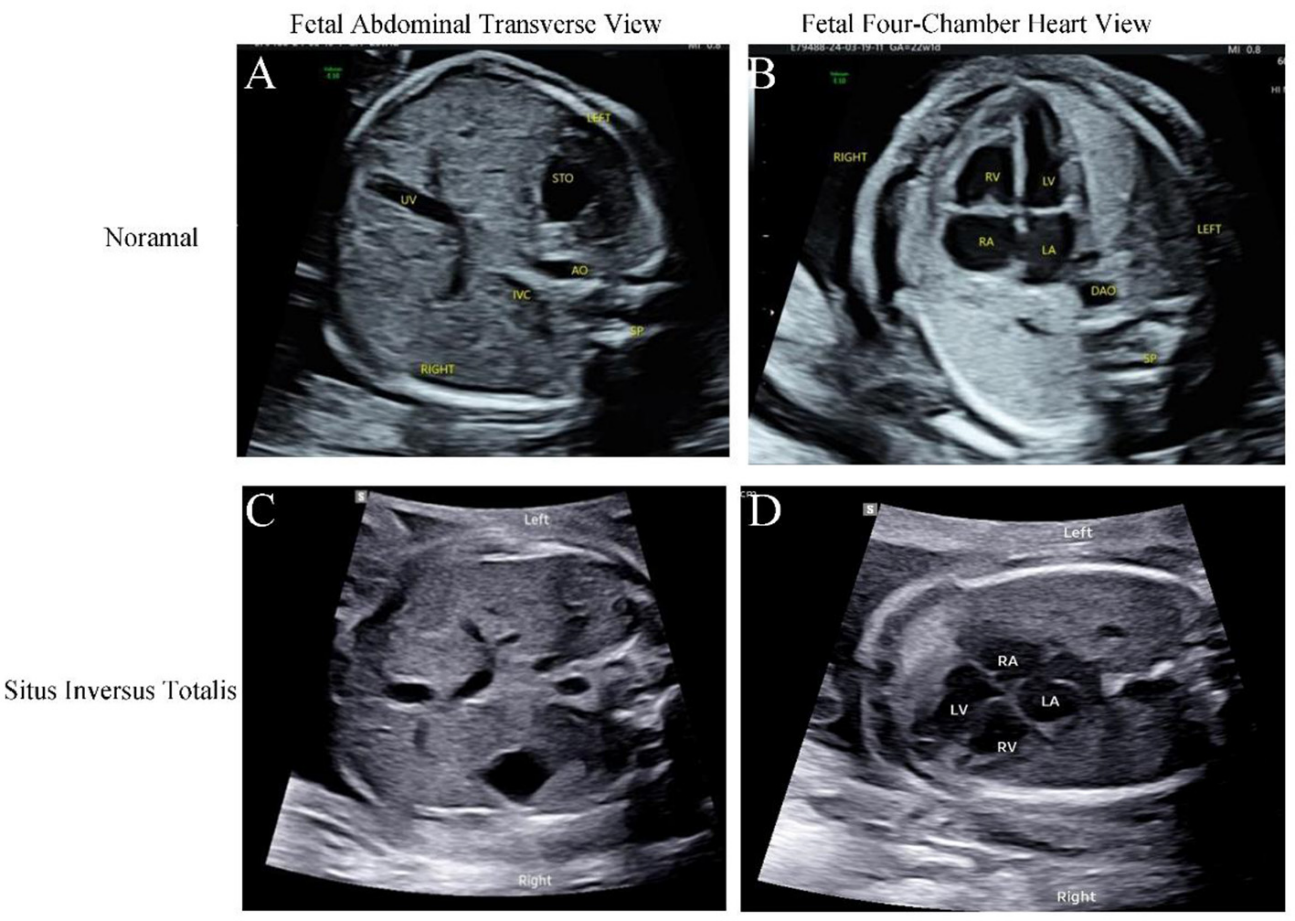
Ultrasonography of a normal fetus: transverse abdominal view **(A)** and four-chamber view of the heart **(B)**; Ultrasonography of fetal situs inversus **(C, D)**. **(C)** Transverse abdominal view showed the stomach bubble located in the right abdomen, the liver in the left abdomen, the abdominal aorta situated right anterior to the spine, and the inferior vena cava located left anterior to the abdominal aorta. **(D)** Four-chamber view showed the heart positioned in the right hemithorax with atrial inversion, right-looping ventricle, and discordant atrioventricular connection.

**Figure 2:**
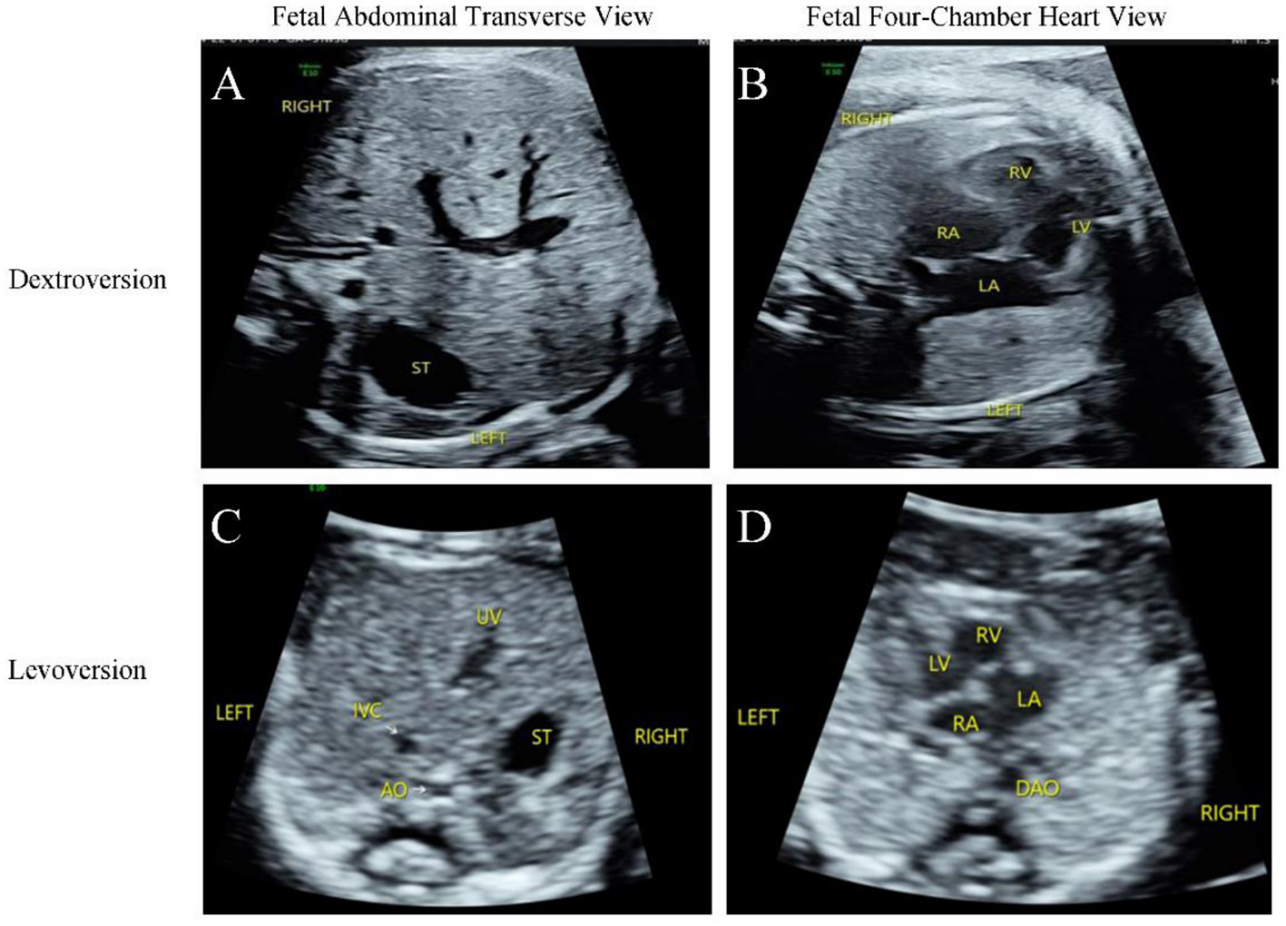
Ultrasonography of fetal dextrocardia **(A, B)**. **(A)** Transverse view of the upper abdomen showed the stomach bubble located in the left abdomen, the abdominal aorta situated left anterior to the spine, and the inferior vena cava located right anterior to the abdominal aorta. **(B)** Four-chamber view demonstrated the heart positioned in the right hemithorax with the cardiac apex pointing to the right, atrial situs solitus, and right-looping ventricle. Ultrasonography of fetal levocardia **(C, D)**. **(C)** Transverse view of the upper abdomen showed visceral situs inversus: the stomach bubble was located in the right abdomen, and the majority of the liver was situated in the left abdomen. **(D)** Four-chamber view during systole revealed the heart located in the left hemithorax with the cardiac apex pointing to the left, atrial inversion, and right-looping ventricle.

**Figure 3:**
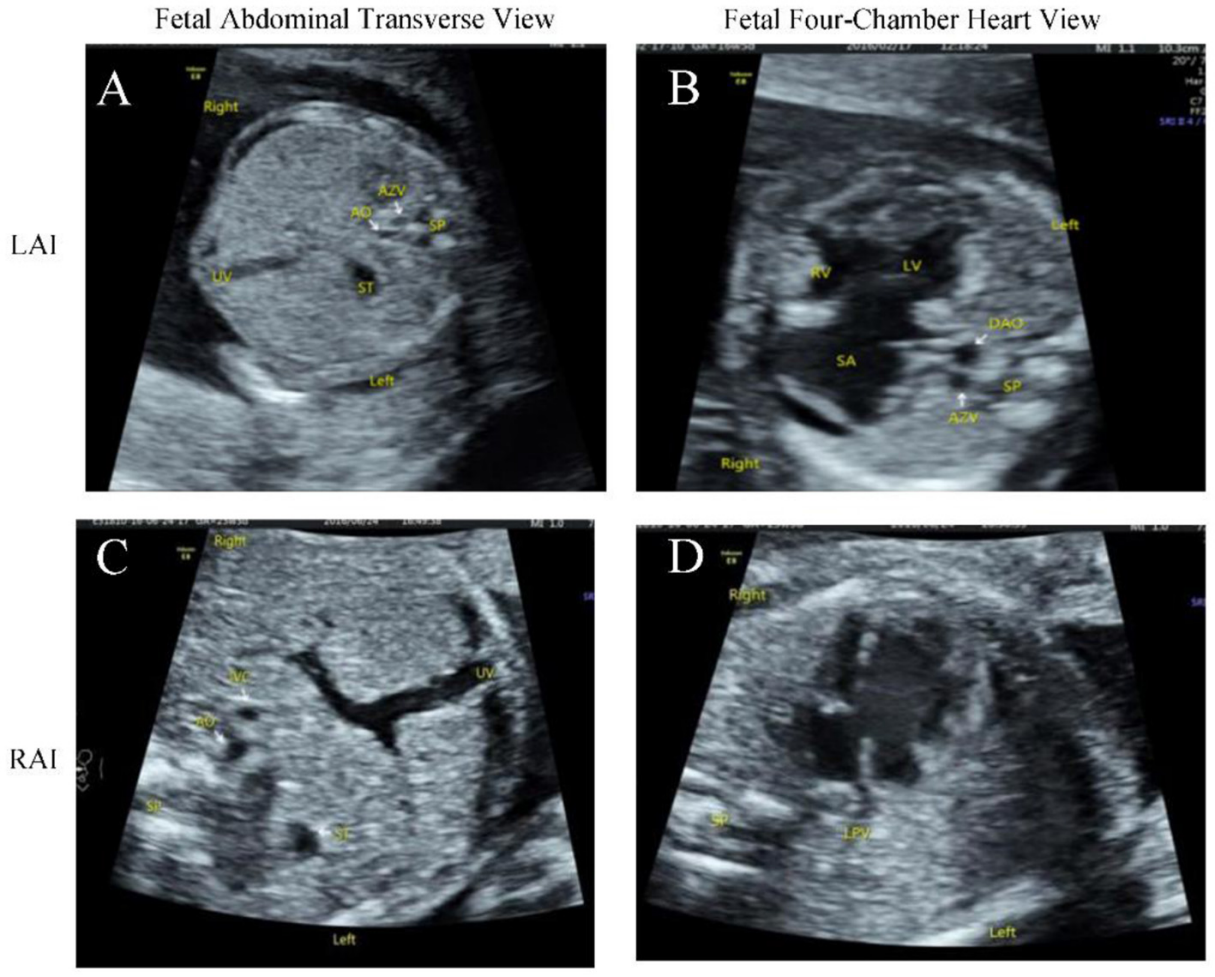
Prenatal ultrasound images of fetal left atrial isomerism (LAI) **(A, B)**. **(A)** Transverse abdominal view showed the stomach bubble located in the left abdomen with absence of the inferior vena cava. The abdominal aorta was positioned anterior to the spine, with a dilated azygos vein visible posterior and to the right of the aorta. **(B)** Four-chamber view demonstrated significant cardiomegaly, a common atrium, and right-looping ventricle. The central crux of the heart was absent with only a common atrioventricular valve observed opening and closing. The descending aorta was located anterior to the spine, with a dilated azygos vein visible posterior and to the right of the aorta. Prenatal ultrasound images of fetal right atrial isomerism (RAI) **(C, D)**: **(C)** Transverse abdominal view showed the stomach bubble in the left abdomen, with the abdominal aorta and inferior vena cava juxtaposed to the right of the spine. The inferior vena cava was located anterior and to the right of the abdominal aorta. **(D)** Four-chamber view showed the heart positioned in the right hemithorax, discordant with the stomach bubble location. A complete atrioventricular septal defect was present, with the ventricles exhibiting a univentricular morphology dominated by right ventricular characteristics.

All 15 hospitals in Fujian Province adopted the same ultrasound diagnostic criteria, and all sonographers were certified for obstetric examinations. A systematic, structured ultrasound scan, including a double check, was conducted. The ultrasound instruments used were Voluson E10 systems (GE Healthcare, Zipf, Austria), with probe frequencies of 4~8 MHz. The diagnostic criteria for fetal situs inversus were: mirror-image relationship of thoracic and abdominal organs compared to normal (Figures 1A–D). The diagnostic criteria for partial situs inversus were: levocardia with abdominal situs inversus, or dextrocardia with normal visceral situs (Figures 2A–D). The diagnostic criteria for heterotaxy syndrome were: for left isomerism, at least two of the following should be present (13): (1) complete atrioventricular septal defect or other structural heart disease; (2) interrupted inferior vena cava with azygos continuation; (3) early fetal heart block; (4) visceral cardiac heterotaxy. For right isomerism, at least two of the following should be present: (1) structural heart disease, namely complete atrioventricular septal defect; (2) juxtaposition of the aorta and inferior vena cava; (3) visceral cardiac heterotaxy (Figures 3A-D).

### Data analysis

Statistical analysis was performed using SPSS software version 20.0. Measurement data were described using counts and proportions. The incidence of fetal laterality disorders was expressed as the number of cases per 10,000 live births. Bar charts were used with the year on the x-axis and the incidence per 10,000 live births on the y-axis, with different colors representing different types of laterality disorders or different regions. The two-proportion Z-test was used to compare the incidence rates across the three periods, and the Chi-square test for trend was used to assess the temporal trend in the prevalence of fetal laterality disorders.

The significance level was set at α = 0.05, and *P* < 0.05 was considered statistically significant. To assess the temporal trends, Poisson regression models were fitted with the period as a categorical predictor and the natural logarithm of total deliveries as an offset. Incidence rate ratios (IRRs) with 95% confidence intervals (CIs) were calculated. Analyses were conducted using SPSS 25.0.

## Results

**Figure 4:**
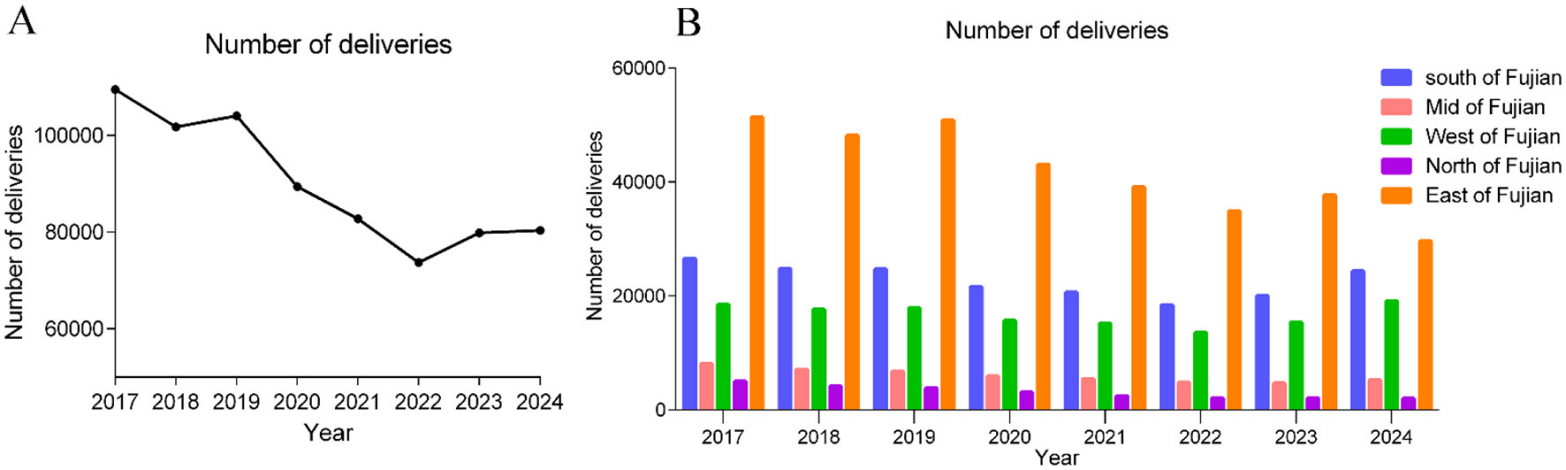
**(A)** Annual number of deliveries in 15 hospitals of Fujian Province. **(B)** Annual number of deliveries by specific region (5 regions) in Fujian Province.

**Figure 5:**
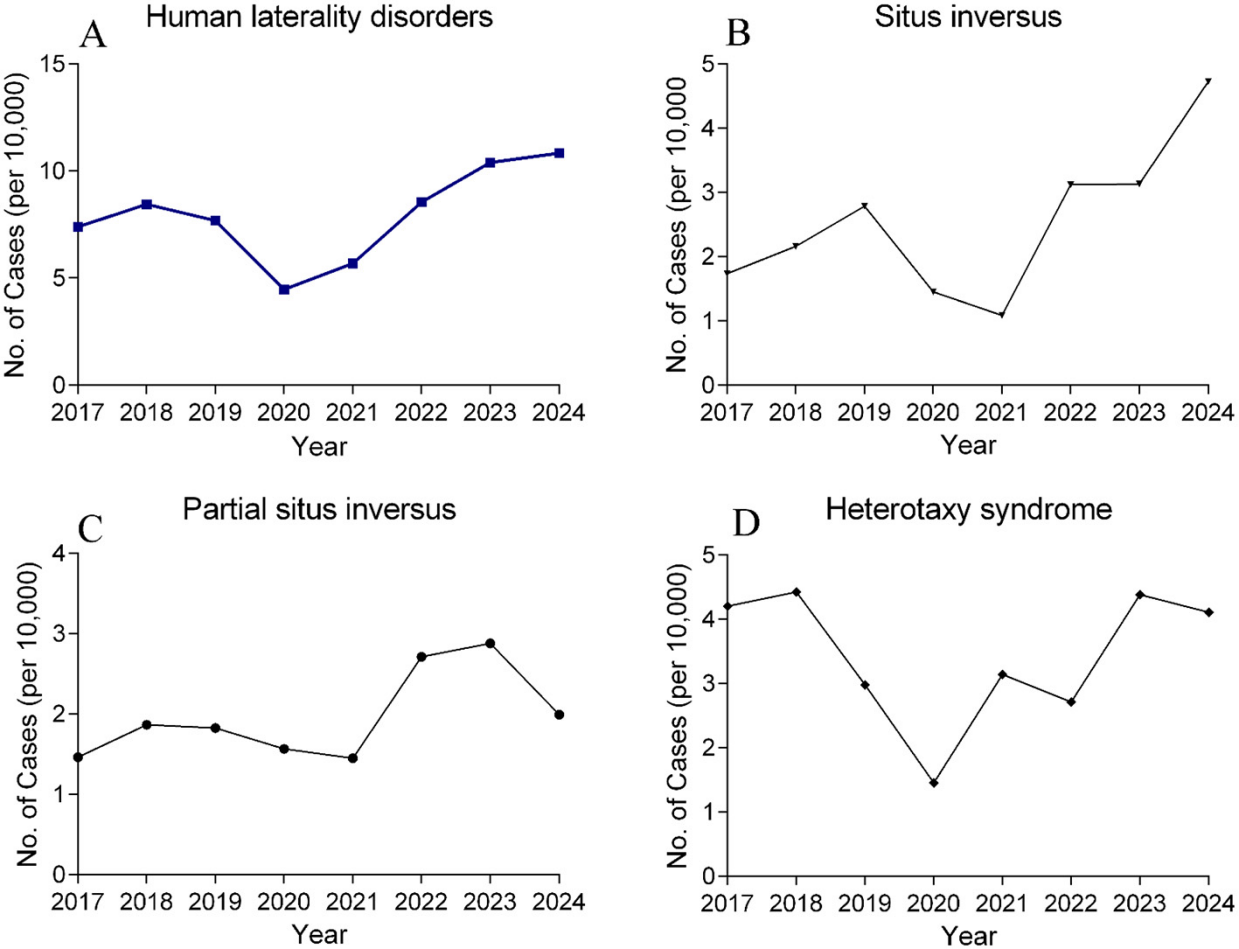
**(A–D)** Incidence of fetal laterality disorders per 10,000 live births by year.

From January 1, 2017, to December 31, 2024, the total number of live births in the 15 hospitals in Fujian Province was 721,562. The annual number of live births is shown in Figure 4A, and the annual number of live births by region in Fujian Province, from highest to lowest, was Eastern Fujian, Southern Fujian, Western Fujian, Central Fujian, and Northern Fujian (Figure 4B). A total of 566 cases were diagnosed with laterality disorders by prenatal ultrasound, including 178 cases of complete situs inversus, 139 cases of partial situs inversus, and 249 cases of heterotaxy syndrome. The annual incidence per 10,000 live births is shown in Figures 5A–D. During the COVID-19 infection period, the prevalence of fetal laterality disorders showed an increasing trend from 2020 to 2024. The number of cases of situs inversus increased most significantly. Further Chi-square test for trend on the prevalence of fetal laterality disorders showed χ^2^ = 7.766, *P* = 0.005, indicating a linear increasing trend in the prevalence of fetal laterality disorders year by year (Table 1). When the years were divided into three stages—pre-COVID-19 infection (2017–2019), Dynamic Zero-COVID Period (2020–2022), and post-pandemic period (2023–2024)—the Chi-square test for trend showed χ^2^ = 5.811, *P* = 0.016, further confirming the linear increasing trend in the prevalence of fetal laterality disorders (Table 2).

**Table 1:** Results of Chi-square test for trend in incidence of fetal lateralization abnormalities.

Year	Total population	Lateralized cases	Non-lateralized cases	χ^2^	*P*
2017	10,9523	81	10,9442	7.766	0.005
2018	10,1787	86	10,1701		
2019	10,4082	79	10,4003		
2020	89,427	40	89,387		
2021	82,771	47	82,724		
2022	73,736	63	73,673		
2023	79,883	83	79,800		
2024	80,353	87	80,266		

**Table 2:** Trend analysis of the incidence of fetal lateralization abnormalities during different stages of COVID-19 infection.

Period	Total population	Lateralized cases	Non-lateralized cases	χ^2^	*P*
Pre-COVID-19 period (2017–2019)	315,392	246	315,146	5.811	0.016
Zero-COVID period (2020–2022)	245,934	150	245,784		
Post-pandemic period (2023–2024)	160,236	170	160,066		

**Figure 6:**
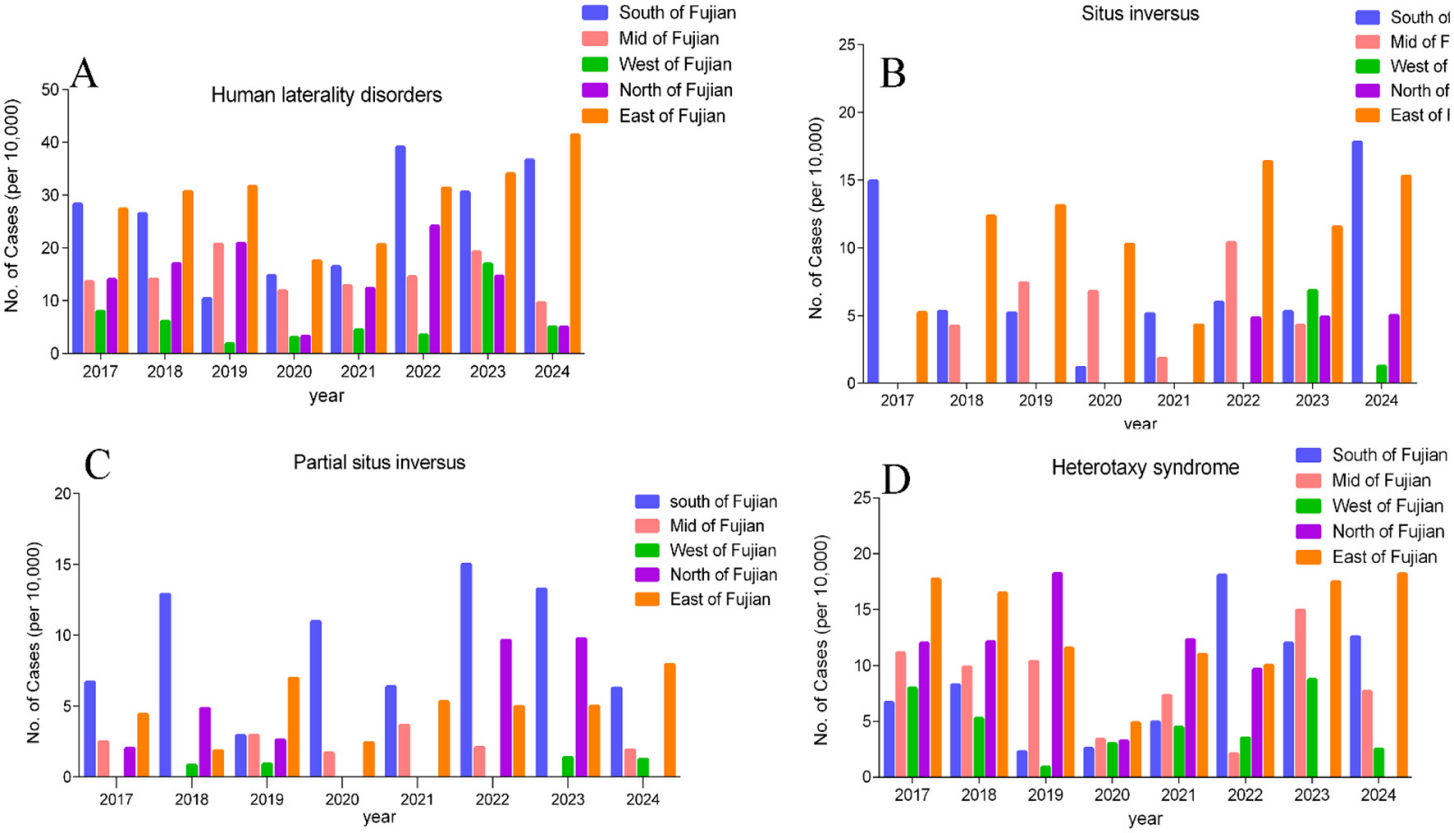
**(A–D)** Incidence of fetal laterality disorders per 10,000 live births by region and year.

Further analysis by time period on the incidence of fetal laterality disorders showed that the average annual incidence during the Dynamic Zero-COVID Period (2020–2022) was 6.10 per 10,000 live births (95% CI: 5.18–7.02), while the average annual incidence during the pre-pandemic period (2017–2019) was 7.83 per 10,000 live births (95% CI: 6.89–8.77). In contrast, during the post-policy adjustment pandemic period (2023–2024), the incidence increased to 10.61 per 10,000 live births (95% CI: 9.07–12.15), which was 1.4 times that of the pre-pandemic period, with no overlap in the confidence intervals compared to both the pre-pandemic and Dynamic Zero-COVID periods (both *P* < 0.001). The average annual incidence of fetal situs inversus across the three periods was 2.2, 1.9, and 3.9 per 10,000 live births, respectively. The incidence during the post-pandemic period (2023–2024) was 1.8 times that of the pre-pandemic period and twice that of the Dynamic Zero-COVID Period (2020–2022). Based on the Figures 6A–D distributed across the five regions, the number of cases of laterality disorders was higher in Southern Fujian and Eastern Fujian in the post-pandemic period (*P* < 0.001), and there is no significant difference in the remaining regions.

To rigorously evaluate the relative risk across different pandemic stages while accounting for variations in total deliveries, a Poisson regression model was performed (Table 3). The model effect test showed that the period variable had a highly significant overall effect on the incidence of complete situs inversus (likelihood ratio chi-square = 24.41, *df* = 2, *P* < 0.001). Setting the Pre-COVID-19 period (2017–2019) as the reference baseline, the Incidence Rate Ratio (IRR) of fetal laterality disorders during the Dynamic Zero-COVID Period (2020–2022) significantly decreased to 0.78 (95% CI: 0.64–0.96, *P* = 0.018). In stark contrast, during the post-pandemic period (2023–2024), the incidence rate surged, demonstrating a significantly higher risk compared to the pre-pandemic baseline, with an IRR of 1.36 (95% CI: 1.12–1.65, *P* = 0.002), indicating a significant increase of approximately 36% compared to the pre-pandemic period. These multivariable modeling results further validate the temporal variations in disease prevalence correspond with the dynamic shifts in national COVID-19 policies.

**Table 3:** Poisson regression analysis of fetal lateralization abnormalities across COVID-19 pandemic periods.

Parameter	B	SE	Wald χ^2^	df	*P*-value	IRR	95% CI for IRR
Intercept	−7.156	0.064	12,598.1	1	< 0.001	0.001	0.001–0.001
Period							
Post-pandemic (2023–2024)	0.308	0.1	9.51	1	0.002	1.36	1.119–1.654
Dynamic zero-COVID (2020–2022)	−0.246	0.104	5.64	1	0.018	0.782	0.638–0.958
Pre-COVID (2017–2019)	0 (ref)	—	—	—	—	1	—

## Discussion

Recent studies in China have reported a higher incidence of fetal situs inversus in pregnant women infected with SARS-CoV-2 in early pregnancy (14, 15). Their studies showed that the number of situs inversus cases in the first seven months of 2023 increased four-fold compared to the average annual incidence from 2014 to 2022 (14). After the lifting of COVID-19 control measures in China, reports from hospitals in Xi'an and Jinan found a significant increase in the diagnosis of situs inversus (16, 17). Our data indicate that the incidence of fetal situs inversus during the post-pandemic period (2023–2024) in Fujian Province was 1.8 times that of the pre-pandemic period and twice that of the Dynamic Zero-COVID Period (2020–2022), further suggesting a potential association between SARS-CoV-2 infection and situs inversus. However, reports from Sweden, Denmark, and Norway showed no sustained increase in the prevalence of situs inversus during the SARS-CoV-2 pandemic period (2020–2022) compared to 2018–2019 (18). Data from four states in the United States also did not find an increase in situs inversus during the SARS-CoV-2 pandemic (11). This may be due to differences in populations and varying policies during the COVID-19 infection period across countries. Situs inversus is a type of abnormal fetal laterality development. We further evaluated the trends in the incidence of fetal visceral laterality disorders before and after the COVID-19 outbreak in 2020 in coastal Fujian Province, China. Our results show that from the emergence of COVID-19 until 2024, the incidence of fetal visceral laterality disorders showed an increasing trend (*P* < 0.05). At the beginning of the COVID-19 pandemic, from 2020 to 2022, due to China's ‘Dynamic Zero-COVID' policy and strict controls, the incidence of fetal visceral laterality disorders decreased and then slowly plateaued compared to the pre-pandemic period. Our results show that the incidence of fetal visceral laterality disorders in 2023–2024 was higher than that in the pre-pandemic period, coinciding with the surge in SARS-CoV-2 infections after China suspended the ‘Zero-COVID' policy. It is estimated that approximately 82% of the population was infected during this period (19). Southern Fujian and Eastern Fujian, as the most populous coastal regions of Fujian Province, also had the highest incidence rates.

Ciliary functional abnormalities, such as primary ciliary dyskinesia, have long been associated with situs inversus (7). It occurs in 25% of patients with complete situs inversus. Although over a hundred genes involved in visceral lateralization have been identified in animal models, the candidate genes for human left-right asymmetry defects are limited. For example, the Nodal gene is responsible for signaling along the left-right axis during embryonic development (20); PITX2, as a downstream gene of Nodal, determines organ asymmetry (21); and Lefty1/2 regulate Nodal expression to ensure left-right differentiation (22). SARS-CoV-2 may directly infect the fetus or indirectly interfere with the expression of these key genes (e.g., Nodal, PITX2) through maternal immune responses, thereby increasing the occurrence of visceral laterality disorders. However, further research is needed to understand how the virus affects these developmental pathways. Moreover, although various gene mutations and chromosomal abnormalities are associated with specific laterality disorders, the relevant clinical features of these disorders can also appear in the absence of these mutations and abnormalities. This does not exclude the potential association between gene and chromosomal abnormalities and visceral heterotaxy; rather, these abnormalities need to be identified. Typical Kartagener syndrome is associated with complete situs inversus and is always characterized by sino-bronchial disease related to ciliary dysfunction. Kartagener syndrome is a well-defined component of primary ciliary dyskinesia syndrome. Primary ciliary dyskinesia occurs in 25% of patients with complete situs inversus. The term Ivemark syndrome refers to patients with right atrial isomerism and asplenia. It is considered an autosomal recessive disorder that primarily occurs in males. It may represent a large subgroup of patients with right atrial isomerism rather than a distinct syndrome. Although the clinical manifestations are quite diverse, visceral heterotaxy is frequently mentioned in syndromic contexts. It is likely that visceral heterotaxy and human laterality disorders exist on a continuum, ranging from syndromic manifestations to sporadic or isolated presentations. Despite extensive research, no specific genes or biochemical markers have been identified that can reliably predict the occurrence of visceral heterotaxy. This may be due to the rarity of these disorders and the incompleteness of research in this field. We believe that the interaction between genetic and non-genetic factors likely leads to the presence and clinical manifestations of visceral heterotaxy and broader human laterality disorders.

The findings of this investigation suggest a potential link between SARS-CoV-2 infection and fetal visceral laterality disorders. However, it is important to recognize that, despite observing this association, one of the study's limitations is its retrospective design. Potential confounding factors cannot be excluded. Secondly, while we tracked SARS-CoV-2 infection in pregnant women during the Dynamic Zero-COVID Period, data on maternal infection during the pandemic period are lacking. Additionally, our study did not include additional basic experiments to explore the underlying mechanisms and pathophysiology. Therefore, future research should focus on confirming our findings and delving into the complex biological mechanisms that may support this association.

## Conclusion

This study found a potential association between maternal SARS-CoV-2 infection and fetal visceral laterality disorders. It highlights the importance for clinicians to maintain vigilance for fetal developmental abnormalities in pregnant women infected with SARS-CoV-2. Furthermore, these findings provide valuable direction for future research on the pathophysiology of visceral laterality disorders.

## Data Availability

The original contributions presented in the study are included in the article/supplementary material, further inquiries can be directed to the corresponding authors.
